# RGST - Rat Gene Symbol Tracker, a database for defining official rat gene symbols

**DOI:** 10.1186/1471-2164-9-29

**Published:** 2008-01-23

**Authors:** Greta Petersen, Fredrik Ståhl

**Affiliations:** 1 Department of Cell and Molecular Biology-Genetics, Göteborgs Universitet, Box 462, SE 40530 Göteborg, Sweden; 2 School of Health Science, University Collage of Borås, SE-501 90 Borås, Sweden

## Abstract

**Background:**

The names of genes are central in describing their function and relationship. However, gene symbols are often a subject of controversy. In addition, the discovery of mammalian genes is now so rapid that a proper use of gene symbol nomenclature rules tends to be overlooked. This is currently the situation in the rat and there is a need for a cohesive and unifying overview of all rat gene symbols in use. Based on the experiences in rat gene symbol curation that we have gained from running the "Ratmap" rat genome database, we have now developed a database that unifies different rat gene naming attempts with the accepted rat gene symbol nomenclature rules.

**Description:**

This paper presents a newly developed database known as RGST (Rat Gene Symbol Tracker). The database contains rat gene symbols from three major sources: the Rat Genome Database (RGD), Ensembl, and NCBI-Gene. All rat symbols are compared with official symbols from orthologous human genes as specified by the Human Gene Nomenclature Committee (HGNC). Based on the outcome of the comparisons, a rat gene symbol may be selected. Rat symbols that do not match a human ortholog undergo a strict procedure of comparisons between the different rat gene sources as well as with the Mouse Genome Database (MGD). For each rat gene this procedure results in an unambiguous gene designation. The designation is presented as a status level that accompanies every rat gene symbol suggested in the database. The status level describes both how a rat symbol was selected, and its validity.

**Conclusion:**

This database fulfils the important need of unifying rat gene symbols into an automatic and cohesive nomenclature system. The RGST database is available directly from the RatMap home page: .

## Background

The rat has long been one of the most widely used animal models in physiology and medicine [1]. The availability of a complete rat genome sequence has now enhanced the rat model even further. In order to facilitate the full use of this genomic resource there has been an extensive exploration of the rat genome with a focus on gene finding.

The number of genes in the rat is estimated to be of the order of 25,000 [2] a similar number to that in both human [3] and mouse [4]. This similarity in the total number of genes is also reflected in the number of orthologous genes; 90% of all rat genes have matches in both human and mouse whereas 80% of all mouse genes have matches in both human and rat [4]. The similarity between mouse, rat and human is the result of a long journey of shared evolution and it is the main reason for the success of rat and mouse as model organisms.

The similarity in gene content is also a cornerstone of rat and mouse gene nomenclature. Gene symbol nomenclature rules for both rat and mouse state that each gene should be assigned according to the gene symbol of its human ortholog, if available [5]. This is a fundamental statement because it reflects the evolutionary meaning embedded in the gene symbols and, most often, it is also referring to function.

When genes are referred to in databases, publications and other methods of communication, the gene symbol should be unambiguously defined to eliminate misinterpretation. The HUGO Gene Nomenclature Committee (HGNC) under the Human Genome Organisation (HUGO) fulfils this need in human by ensuring that only a single symbol is approved for each gene [6]. Thus, by using the human as a template for gene naming the gene symbols in mouse and rat becomes integrated into the human gene nomenclature system.

Ideally, the gene symbol nomenclature rules among mammals may serve as a means for easily communicating information across species. Adopting human gene symbols for orthologous genes in other mammals is both a rational and a practical way to create a coherent and meaningful gene nomenclature.

There are two databases, RatMap [7] and RGD [2], which aim to provide a central repository of rat gene symbols. Both RatMap and RGD are acclaimed providers of official rat gene symbols as specified by the Rat Genome and Nomenclature Committee [8]. They differ in many ways from other databases of rat genome data, such as Ensembl [9] and NCBI-Gene [10]. These differences may include usage of different gene symbols, different references to human orthologs, genes that are not uniquely identified, and, on occasion, different chromosomal positions [11].

It should, however, be emphasized, that the problem of nomenclature inconsistencies is not exclusive to the rat and goes well beyond the scope of gene symbols. Although nomenclature rules already exist for a large number of species, they are seldom fully implemented throughout the scientific community and between individual databases. For example, Fundel and Zimmel [12], who compiled gene and protein name dictionaries for yeast, fly, mouse, rat and human, found that the number of synonyms varied significantly between individual databases for a given species, as did the degree of synonym ambiguity between the species.

The recent rapid increases in rat gene discovery have put a great deal of pressure on manual curation. About two thirds of all known rat genes have gene identifiers that differ between databases or are not in full agreement with the present gene symbol nomenclature rules. In our opinion, there is a need for a function that unifies all rat genes into a single and cohesive nomenclature system. With limited manual curation available, the solution must be an automatic function that deals with rat gene symbol issues in a way consistent with the established rat gene symbol nomenclature rules.

In this paper we present a database called Rat Gene Symbol Tracker (RGST). This database browses through gene data from Ensembl, NCBI and RGD, tracks down inconsistent positional and gene symbol data, automatically implements the major rat gene symbol nomenclature rules and suggests a single gene symbol. Equally importantly, the system also assigns a status level, which classifies the validity of each gene symbol and genomic position. Suggested symbols along with status level and accompanying information on sources are made available from the RatMap web site [13]. Furthermore, the RGST system has been adopted as the core facility of the RatMap database and here serves as a quick and easy platform for providing a conclusive overview of rat genes and rat gene symbols.

## Construction and Content

The objective of this project was to create a database that assigns a conclusive gene symbol for each established and predicted gene position in rat, following the rat gene symbol nomenclature rules as described by the RGNC [14]. The procedure involves the assembling of rat gene data, human gene data, rat-human gene orthology data and mouse-rat orthology data. The data is obtained from Ensembl [15], NCBI [16], the Rat Genome Database (RGD) [17], Mouse Genome Informatics (MGI) [18], and the HUGO Gene Nomenclature Committee (HGNC) [19]. All data is stored and interconnected within a local MySQL database. Linking between objects obtained from the various databases is based on common Ids; Ensembl Gene ID, NCBI-Gene ID, RGD ID, and MGI ID.

The automatic naming process is based on the following three steps: 1) where possible, the human gene symbol is used as a template; 2) if this is not possible, a template is sought first in RGD and then in other sources of rat gene symbols; and 3) if neither of the previous steps proves fruitful the mouse gene symbol is used as a template. A status description is attached to each gene symbol declaring the validity of the symbol and how it was determined. Next, a "cleaning" step is undertaken, to eliminate inadequate or duplicated symbols. Finally, the resulting symbol is checked against a standard set of gene symbol nomenclature rules.

### Rat genes assigned according to HUGO

According to the rat gene symbol nomenclature rules, rat genes orthologous to human genes should adopt the human symbol (with some syntax changes). The RGST gene symbol pipeline starts by dividing the rat genes into two groups: rat genes with and without an orthology to human genes. The rat-human gene orthology data is obtained from the Ensembl Gene Orthology/Paralogy prediction method but only "ortholog_one2one" (formerly known as "best reciprocal hits") are used.

If a human HUGO symbol agrees with at least one known rat symbol the rat gene symbol is given **status H1**, if not, the rat symbol is assigned **status H2**.

### Rat genes assigned according to RGD

Rat genes that have a human ortholog without a HUGO symbol, as well as rat genes without a human ortholog, are checked for gene symbol agreement between the three rat genome databases RGD, Ensembl, and NCBI-Gene. If all three databases agree the rat symbol is assigned **status R1**. If there is disagreement, but an RGD symbol exists, this symbol is used and the status is set to **R2**, regardless of the symbols used in the other two databases.

### Rat genes assigned according to other available rat symbol

Rat genes that fulfil the conditions described in 4.2 but have no RGD symbol and two identical, or a single, rat symbol(s), keep their original symbol and are temporarily assigned status R3. For this status to be retained, the resulting rat gene symbol must not agree with the symbol of a putative orthologous mouse gene as described below.

### Rat genes assigned according to MGD

Regardless of their status in Ensembl and NCBI-Gene, all rat genes that fulfil the conditions described in 4.2 but have no RGD symbol are checked against orthologous mouse genes [20]. The rat-mouse gene orthology data is obtained from the Ensembl Gene Orthology/Paralogy prediction method pipeline but only "ortholog_one2one" (formerly known as"best reciprocal hits") are used.

Rat genes that have a mouse ortholog with an established symbol are named according to the mouse gene symbol. They are assigned **status M1 **if an identical rat gene symbol has already been found as described above, and **status M2 **otherwise.

### Manual considerations

Rat genes that have no RGD symbol and two non-identical symbols in the other databases, or that have no rat symbol at all and also no orthologous mouse gene symbol are assigned a provisional accession ID as an identifier. These genes are given the **status IS **(illegitimate symbol) and are left for manual consideration.

Rat genes that have rat symbols that disagree with each other and with the symbol of an orthologous mouse gene are also assigned a provisional accession ID as identifier. These genes are also given the **status IS **(illegitimate symbol) and are left for manual consideration.

### Nomenclature review

According to the rat gene symbol nomenclature rules, a rat gene symbol should be short, preferably consisting of between 3–5 letters, and never of more than ten characters. All characters must be Roman letters or Arabic numbers. Other characters, such as Greek letters, Roman symbols or hyphens are usually not allowed. It is desirable that the initial character in the gene symbol is equivalent to the first letter in the corresponding gene name. However, this does not mean that the characters in the gene symbol need to follow the word order of the gene name. When gene symbols are referred to in a text they should normally be written in italics and the first letter in the gene symbol should be capitalised. The characters following the first one should be lower case letters or numbers [14].

In order to make sure that the basic rat gene symbol nomenclature rules are followed an automatic control is built into RGST. Thus, all rat symbols are checked to ensure that they do not contain more than ten characters and that they consist of a capital letter followed by lower cases or numbers. They should also not have the word "rat" at either end or beginning as a reference to the species. However, "rat" is permissible where it is dictated by the gene name or description, for example, in the case of Lrat, which is an acronym for Lecithin retinol acyltransferase. Furthermore, specific symbols such as "@", "_" ".", and so on are not allowed, and are actively retrieved within database as well gene symbols that are accessions ID:s

If a gene symbol is found to be illegitimate, the symbol is still used together with its originally assigned status, but it is given the additional **status IL **(illegitimate nomenclature). These gene symbols are left for manual consideration.

### Human ORFs

Specific attention is paid to the naming system of the human ORFs, since they are named according to their human chromosomal position. Where an open reading frame on a human chromosome has been given a HUGO symbol, for example "C10orf46", according to the Guidelines for Human Gene Nomenclature [21], the orthologous rat symbol in RGST would be named analogously; "C1H10orf46" in this case (Chromosome 1 open reading frame, human C10orf46).

## Utility and Discussion

To be able to talk about a gene, it is necessary to know its name. This may seem to be a simple task, but with the tremendous increase in known genes we have seen in recent years, the naming of genes has become far from simple. Gene symbol nomenclature rules for most species are intended to guide scientists and curators in creating gene symbols that are meaningful, both in themselves but also in relation to other gene symbols; that share a general structure with all other symbols within the species; and that can be compared to orthologous genes in other species. However, an overview of current gene symbol nomenclature in the rat (Sept 12, 2007) reveals that approximately 12000 genes have been given contradictory symbols from different sources. Only around 7 800 rat genes can be said to fully satisfy the rat gene symbol nomenclature rules, and roughly 7400 genes have no real symbol at all.

### Rat Gene Symbol Tracker (RGST) – overview

At present, the rat gene symbol nomenclature rules are maintained on a day-by-day basis by the RGD [17]. However, the rapid increase in gene finding has placed an overwhelming workload on manual curators. Based on longstanding experience in rat gene symbol nomenclature, RatMap has now released a new database known as RGST (Rat Gene Symbol Tracker). RGST automatically implements the most important rat gene symbol nomenclature rules on rat gene symbols obtained from RGD [17], Ensembl [15], and NCBI [16]. Rat gene symbols are compared with firstly human symbols approved by HGNC [19] and secondly mouse gene symbols obtained from MGI [18]. These procedures result in the designation of conclusive gene symbols.

For each gene symbol acknowledged by RGST, the outcome of this characterization is a conclusive rat gene symbol, which is presented together with a status level that clearly defines the validity, and to some extent the meaning, of the symbol used. That is, RGST does not simply name genes; it also categorizes gene symbols in a systematic way according to the basic rat gene symbol nomenclature rules. Thus, the names of the genes are seen as the logical outcome of the system, and hence as much attention should be paid to the status level accompanying the symbol as to the symbol itself.

RGST is a resource that can be used by the individual researcher for finding information on rat gene symbols as well as their reliability. RGST is also a database that may be useful for database curators in retrieving rat gene symbols that need to be manually curated.

### RGST – function

The RGST database forms the core of the RatMap website [13], which provides several ways to search for a rat gene symbol. Firstly, a specific genomic region can be queried by entering chromosome number and/or base pair position. Since RGST integrates rat and human gene homology data, it is also possible to find rat gene symbols by querying a human genomic region.

Rat gene symbols can also be found by querying for any known rat gene symbol, official or obsolete. Queries can also be made on accession IDs from RatMap, Ensembl, NCBI Entrez-Gene, RGD, and the HUGO Nomenclature database. For any given query, the user obtains a list that contains information on gene symbols that have been approved by the RGST database. Each approved gene symbol is accompanied by a status description, as summarized in Table 1.

**Table 1:** Status level definitions. Status level definitions accompanying each gene symbol verified by RGST.

H1	Rat gene that has a human ortholog with an established human HUGO symbol that is identical with at least one of the rat gene symbols from RGD, Ensembl, or NCBI. The human HUGO symbol is used as a rat gene symbol reference.
H2	Rat gene that has a human ortholog with an established human HUGO symbol that is inconsistent with rat gene symbol(s) from RGD, Ensembl, and NCBI. The human HUGO symbol is used as a rat gene symbol reference.
R1	Rat gene that does not have a human HUGO symbol as a reference. RGD symbol available, and in agreement with both Ensembl and NCBI (if available). The RGD symbol is used as a rat gene symbol reference.
R2	Rat gene that does not have a HUGO symbol as a reference. RGD symbol available, but in disagreement with Ensembl and/or NCBI. The RGD symbol is used as a rat gene symbol reference.
R3	Rat gene that does not have a human HUGO symbol as a reference. No RGD symbol available. No mouse MGI symbol available. Identical symbol in Ensembl and NCBI, or symbol only present in one of these two datasets; this symbol is used as a rat gene symbol reference.
M1	Rat gene that does not have a human HUGO symbol as a reference. No RGD symbol available. Mouse MGI symbol identical with at least one symbol from Ensembl or NCBI; this symbol is used as a rat gene symbol reference.
M2	Rat gene that does not have a human HUGO symbol as a reference. No RGD symbol available. Mouse MGI symbol is used as a rat gene symbol reference.
IL	Rat gene that cannot be categorized in any of the above status levels. Reserved for manual consideration.
IS	Where a gene symbol is not in accordance with rat gene nomenclature rules, its status is given the suffix IS.

Rat gene symbols used in RGD, NCBI-Gen,e and Ensembl are presented as well as symbols from orthologous genes in human (HUGO) and mouse (MGI). Direct URL links are provided for all these databases.

Clicking on the Approved Gene Symbol provides the user with more detailed information on symbol usage, functional description, and chromosomal position.

### Status levels

#### H1 and H2

**Figure 1:**
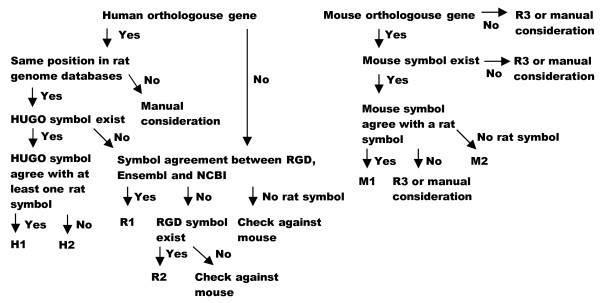
**Status level process**. Overview of the status level process.

For all rat genes that have an orthologous human gene with an accepted HUGO symbol, and for which at least one rat genome database has already adopted the same symbol, we have assigned a status level called H1 (Fig 1). We distinguish the H1 symbols from those where a HUGO symbol exists but there is no matching rat symbol; these latter genes still adopt the HUGO symbol but are given the status H2 (Fig 1). The reason for making this distinction is that gene symbols are supposed to convey the character or function of a gene, and whereas status H1 strongly suggests that this is the case, status H2 indicates that it may not be so [6]. This is of course a very crude way of estimating the presence of potential differences in gene function between species, and it is possible that a future development of the database, or a compilation utilizing manual curation, could refine H2 status to only represent genes with truly different functions in human and rat.

#### R1, R2, R3

If no HUGO symbol exists, a rat symbol is generated according to a systematic process which results in status levels R1, R2, and R3 (Fig 1).

In cases where RGD alone or together with Ensembl, and NCBI-Gene, if available, use the same symbol for a given gene, this symbol is used as the RGST symbol, and is given status level R1. Where the three rat genome databases disagree, the RGD symbol is used, since RGD is the official rat gene symbol nomenclature site. Discrepancies in symbol usage between rat genome databases may reflect contradictory views of gene function, and so the R2 status is used to draw attention to such possibilities.

R3 status is given to symbols that do not appear in RGD, but that are identical between Ensembl and NCBI-Gene, or that only appear in one of the two databases, and for which there is no additional information from either human or mouse. Naturally, this is not a very satisfactory situation, and hence R3 status indicates that the symbol may change over time.

#### M1 M2

Where neither HUGO symbol nor RGD symbol exists, rat symbols are compared to orthologous mouse genes. The principle is that the mouse gene symbol will act as a template for the rat gene symbol. These gene symbols are assigned status M1 and thus rely on the assumption that genes orthologous between rat and mouse share function. Of course, the guiding mouse symbols have no HUGO counterpart, but use of the same symbol in mouse and rat, where applicable, will decrease the number of different symbols, leading to less confusion overall.

### Ranking of the status levels

Certainly H1 is the highest ranked status, and ideally all rat symbols will eventually achieve this status. That is to say, when RGD officially adopts a symbol that is already accepted by HUGO for the orthologous human gene, the rat symbol reaches full acceptability. At present, 6131 (Sept 12, 2007) rat genes have status H1.

The second-ranked status is H2. Every rat gene is supposed to adopt the human symbol, and this is in fact the case with H2 symbols; however, if differences in symbol usage between human and rat do indeed reflect a functional difference, then this must be made apparent in some way. Ideally, each individual case should be examined and judged by curators. However, since there are currently 6592 (sept 12, 2007) rat gene symbols with H2 status, we will probably have to live with the existence of this status for quite some time. In addition, genes that have a distinct function in rat and human will probably never achieve status H1; in fact, it may be worth considering an alternative naming system for genes with multiple functions.

R1, R2, and R3 all denote symbols that use rat gene names as a primary source. Where possible, these genes should eventually move to H1 or H2 status when an orthologous human gene becomes established. However, there are a few genes that are found only in rat, such as certain olfactory genes. Naturally, it will never be possible to assign these genes a corresponding human symbol, and so their status will remain unchanged. The numbers of rat gene symbols with status of R1, R2, and R3 are; 4861, 1355, and 1385, respectively (Sept 12, 2007).

Finally, status levels M1 and M2 are the lowest-ranked, since rat gene symbols are not supposed to follow the mouse; naming a rat gene according to an orthologous mouse gene is, in principle, to be considered an emergency measure.

### Manual consideration

Some cases, such as contradictory rat symbols other than RGD-symbols (Fig. 1), cannot be resolved automatically within the present database and are thus left for manual consideration. Resolution of the symbols and status of these genes should be, in our opinion, of the highest priority for manual curators.

### Similar databases

Recently, HGNC released a similar database to RGST, HCOP [22], and it may be worthwhile to compare the two. The main purpose of the HCOP resource is to provide a useful method to integrate, compare and access a variety of disparate sources of human orthology data. However, this is not always so simple. For instance, the human gene ABO (Entrez ID:28) is presented with two predicted orthologs (paralogs?) in rat: Abo (Entrez ID:65270, RGD ID:628609) and Abo-predicted (Entrez ID:296504, RGD ID:131152). HCOP provides information on possible orthologs between rat and human, but no clear information on correct symbol usage in the rat. In RGST, the Abo gene symbol is classified as R1 whereas the Abo-predicted gene is automatically classified as requiring manual consideration, since they are considered to be paralogs.

In brief, this illustrates the different usage and functions of HCOP and RGST. Whereas HCOP gives as much homology data as possible, RGST is restricted to clear cases of gene orthology. Furthermore, RGST takes the gene orthology as a starting point for assigning a conclusive rat gene symbol accompanied by a status description. In addition, RGST also deals with rat genes that have no orthologs in either human or mouse. Thus, both HCOP and RGST use orthology as a basis for their function, whereas HCOP provides as much orthology data as possible, RGST defines gene symbols based on only clear-cut cases of orthology.

## Conclusion

We believe that RGST fills an important function by automatically assigning conclusive symbols to rat genes in accordance with the rat gene symbol nomenclature rules. Symbol discrepancies and similarities between different databases can easily be found, and the original data quickly retrieved. RGST also brings robustness to the assigned symbol by associating each symbol with a status description, which provides the user with helpful hints on gene function and orthologies.

Furthermore, RGST retrieves and lists genes with several kinds of inconsistent or uncertain data. This listing, together with the accompanying status descriptions, can be used for directing curation to the most urgent needs in naming rat genes.

## Availability and requirements

Project name: Rat Genome Symbol Tracker;

Project home page: ;

Operating system(s): Platform independent;

Programming language: none;

License: no restriction;

Any restrictions to use by non-academics: no restriction.

## Authors' contributions

GP carried out the programming, design and implementation of the RGST database. FS conceived of the study, and participated in its design and coordination and helped to draft the manuscript. All authors read and approved the final manuscript.
